# Overtraining Strengthens the Visual Discrimination Memory Trace Outside the Hippocampus in Male Rats

**DOI:** 10.3389/fnbeh.2021.768552

**Published:** 2021-11-15

**Authors:** Hugo Lehmann, Morgan G. Stykel, Melissa J. Glenn

**Affiliations:** ^1^Department of Psychology, Trent University, Peterborough, ON, Canada; ^2^Department of Psychology, Colby College, Waterville, MA, United States

**Keywords:** retrograde amnesia, lesion, consolidation, hippocampus, rat

## Abstract

The hippocampus (HPC) may compete with other memory systems when establishing a representation, a process termed overshadowing. However, this overshadowing may be mitigated by repeated learning episodes, making a memory resistant to post-training hippocampal damage. In the current study, we examined this overshadowing process for a hippocampal-dependent visual discrimination memory in rats. In Experiment 1, male rats were trained to criterion (80% accuracy on two consecutive days) on a visual discrimination and then given 50 additional trials distributed over 5 days or 10 weeks. Regardless of this additional learning, extensive damage to the HPC caused retrograde amnesia for the visual discrimination, suggesting that the memory remained hippocampal-dependent. In Experiment 2, rats received hippocampal damage before learning and required approximately twice as many trials to acquire the visual discrimination as control rats, suggesting that, when the overshadowing or competition is removed, the non-hippocampal memory systems only slowly acquires the discrimination. In Experiment 3, increasing the additional learning beyond criterion by 230 trials, the amount needed in Experiment 2 to train the non-hippocampal systems in absence of competition, successfully prevented the retrograde amnesic effects of post-training hippocampal damage. Combined, the findings suggest that a visual discrimination memory trace can be strengthened in non-hippocampal systems with overtraining and become independent of the HPC.

## Introduction

Damage to the hippocampus (HPC) can cause retrograde amnesia for memories that are termed episodic or declarative, yet the vulnerability of these memories to HPC damage is not ubiquitous (Scoville and Milner, 1957; Rempel-Clower et al., 1996). For instance, there is evidence suggesting that HPC damage is more likely to spare older or remote memories (Scoville and Milner, 1957; Kim and Fanselow, 1992). This phenomenon has been accounted for by long-term systems consolidation theories, which propose gradual strengthening of the representation in neocortical structures resulting in a decreased or differential contribution of the HPC to the recall process (Squire, 1992; Alvarez and Squire, 1994; Squire and Alvarez, 1995; Nadel and Moscovitch, 1997; Frankland and Bontempi, 2005; Winocur et al., 2010; Sekeres et al., 2018). The distributed reinstatement theory (DRT), however, introduced an alternate account to this time-dependent change; it states that re-experiencing the learning episode can be key in making a memory become established in other networks and more resistant to HPC damage, whereas time is a mere cofactor of the process (Sutherland et al., 2010).

The DRT is founded on three main tenets: (1) that there are multiple memory systems, (2) that for some forms of memory the HPC overshadows other systems, and (3) that the overshadowing can be abated with additional learning (Sutherland et al., 2010). The perspective that there are multiple neural memory systems is not new and is widely accepted (Milner, 1959; Sherry and Schacter, 1987; Packard et al., 1989; Packard and McGaugh, 1996; Squire and Zola, 1996; Nadel and Moscovitch, 1997; White and McDonald, 2002). Less described is the idea that the HPC may overshadow other memory systems, which is supported by the evidence that HPC damage induced *after* but not *before* learning causes amnesia for the same type of memory (see Sutherland et al., 2010; Lee et al., 2016). For example, HPC damage after contextual fear conditioning causes profound retrograde amnesia in rats, suggesting that the memory is normally dependent on the HPC (Lehmann et al., 2007, 2013; Sutherland et al., 2008; Broadbent and Clark, 2013; Sparks et al., 2013). The same damage, however, does not impair the rats’ ability to acquire and retain a new contextual fear memory (i.e., no anterograde amnesia), suggesting that a non-HPC memory system is now supporting the memory (Maren et al., 1997; Wiltgen et al., 2006; Lehmann et al., 2009). If other systems can acquire and support a memory in the absence of the HPC, then why do they not do so when the HPC is present at the time of learning? This can be parsimoniously answered by an overshadowing process between the HPC and non-HPC systems, in which the HPC prevents the other systems from acquiring an independent memory (Maren et al., 1997; Fanselow and Poulos, 2004; Driscoll et al., 2005; Lehmann et al., 2006; Sutherland et al., 2006; Lee et al., 2016). According to the DRT and despite the HPC overshadowing, other systems can come to support the memory if there are sufficient additional opportunities to reinstate the memory (Sutherland et al., 2010; Sutherland and Lehmann, 2011). Evidence for the DRT has come from contextual fear conditioning experiments in which distributed fear conditioning episodes prevented the typical retrograde amnesic effects of complete HPC damage (Lehmann et al., 2009). For example, HPC damage after a single conditioning session with 12 context-shock pairings eliminated the memory. Yet, in the same study, complete HPC damage after the same number of context-shock pairings distributed across 11 conditioning sessions prevented the amnesia. Hence, distributing the learning made the memory stronger in other systems as it no longer required the HPC for expression.

The DRT and its postulated process in making a memory become HPC independent has seldom been investigated beyond that of context fear memory. Therefore, we tested the generalizability of the DRT by examining whether additional learning also make a visual discrimination memory become resistant to HPC damage. We selected this type of memory because several memory systems contribute to visual discrimination memory in rats and the HPC overshadows these other systems. Specifically, evidence suggests that damage to the HPC after learning caused severe retrograde amnesia for a visual discrimination memory, but that the same rats were able to relearn the discrimination following the HPC damage (Sutherland et al., 2001; Driscoll et al., 2005; Epp et al., 2008). Given that non-HPC memory systems can acquire and support a visual discrimination memory in the absence of the HPC and in accordance with the DRT, we predicted that overtraining a visual discrimination memory would mitigate the retrograde amnesic effects of HPC damage.

## Experiment 1

The present experiment examined whether strengthening a visual discrimination memory, once acquired, using additional learning trials and distributing them over several weeks would increase the likelihood of the memory becoming independent of the HPC. Specifically, rats were trained to criterion on a visual discrimination (80% accuracy on two consecutive days) and then given 50 additional training trials. This number approximates the number of trials that others have suggested is needed to train the non-HPC system in the absence of overshadowing (Epp et al., 2008). Also, in the present experiment, the additional learning was either massed within 5 days (10 trials per day for 5 days; Massed) or distributed across 10 weeks (five trials in one session per week for 10 weeks; Distributed). Because distributed learning is well known to establish stronger memories (Wagner et al., 1973; Bahrick and Phelphs, 1987), the Distributed condition was hypothesized to be the most likely to make the visual discrimination memory become HPC independent; the Massed condition served as a control for the overall number of learning trials and the time interval between learning the discrimination to criterion and the HPC damage.

### Methods

#### Subjects

All procedures were approved by the Trent University Animal Care Committee, which follows the guidelines set by the Canadian Council on Animal Care. The subjects were 38 male Long Evans rats weighing ∼300 g at the beginning of behavioral training. The rats were housed in groups of two in standard laboratory cages and maintained on a 12:12-h light-dark cycle (lights on at 0700 h). Each rat received 25–30 g of rat chow daily and had access to water *ad libitum*.

#### Apparatus

**FIGURE 1:**
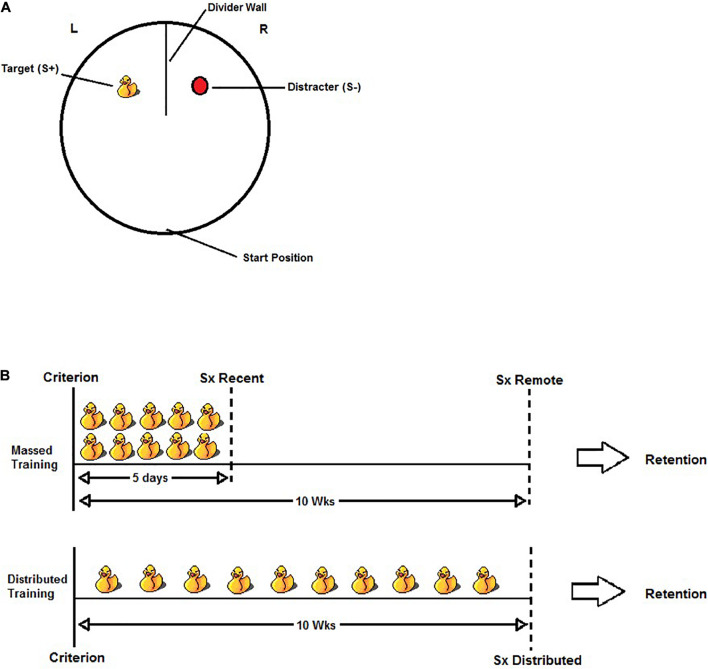
**(A)** Illustration of the visual discrimination task. Rats were released from the start position and given a maximum of 60 s to swim to the S+ and escape from the water. A trial was considered correct if the rat swam to the S+ and climbed onto the hidden platform without first entering the S– quadrant. A trial was considered incorrect if the rat’s entire body (excluding tail) entered the S– quadrant. The S+ was counterbalanced within groups such that the rubber duck was the S+ for half the rats and the red buoy was the S+ for the other half. The location of S+ was also pseudo-randomly counterbalanced across trials within a swim session so that the rats would learn to swim to the S+ and not a discrete quadrant of the pool. **(B)** Illustration of the experimental design for the massed training condition (top) and the distributed training condition (bottom). After reaching criterion (defined as >80% accuracy over two consecutive days), all rats were given an additional 50 discrimination trials (each duck represents five trials). In the Massed Training condition, the rats received 50 additional trials over 5 days (10 trials per day). The Recent group received either sham or HPC surgery (Sx) 5–10 days following the last day of training, whereas the Remote group received sham or HPC surgery 10 weeks following the last day of training. In the Distributed training condition, the rats received the additional 50 training trials distributed over 10 weeks (five trials in one session per week). The distributed training group received sham or HPC surgery 5–10 days following their last day of training (approximately 10–11 weeks after reaching criterion). In all cases, the rats were given a minimum of 10 days recovery before testing for retention.

The visual discrimination apparatus consisted of a circular pool (140 cm in diameter and 60 cm high) filled with water (21 ± 1°C) to a depth of 32 cm. The pool was located toward the center of a standard laboratory behavioral testing room and was not isolated from the room’s visible cues (e.g., door, shelving, etc.). The water was made opaque using powdered skim milk. A wall (51.5 cm wide and 61 cm high), made of white corrugated plastic, was positioned at the north end of the pool to form two equal-sized compartments, each containing a discrimination stimulus in the center. The discrimination stimuli were a yellow rubber duck (Length × Width × Height; 10 × 10 × 12 cm) and an oval-shaped red buoy (9 × 9 × 12 cm). The stimulus that predicted escape from the water (S+) was attached to a submerged platform (15 cm in diameter and 2 cm below the water surface). The other stimulus (S−) did not predict escape as it was not attached to a platform. An illustration of the apparatus and task is found in Figure 1A.

#### Procedures

##### Pre-surgical training

###### Criterion training

The rats, one at a time, were transported to the testing room in a white opaque plastic bucket, which was used as a holding cage between trials. The rats were given 10 discrimination trials per day; for half the rats the S+ was the rubber duck and for the other half it was the red buoy. For all trials, the rats were released from the same starting position facing the southern wall of the pool and given a maximum of 60 s to escape from the water onto the platform hidden below the S+. After escaping from the water, the rats were given an additional 10 s on the platform prior to being removed from the pool and placed in the holding cage. If the rats did not escape the water onto the platform within 60 s, then they were guided to the escape and given 10 s on it before being removed from the pool. The interval between trials was approximately 1 min. The location of the S+ was counterbalanced pseudo-randomly across trials within a session in order for the rats to learn that the S+ predicted escape from the water. This also ensured that spatial cues in the room were not predictive of the platform location and that directional swim strategies (e.g., swim right) would also be unsuccessful to perform the discrimination.

A trial was considered correct if the rat swam directly to the S+ and climbed onto the platform without entering the S− quadrant. A trial was considered incorrect if the rat’s entire torso passed the dividing wall into the S− quadrant, but the trial was permitted to continue until the escape platform was found or the maximum trial duration. Training continued until the rats reached a criterion of 80% (8 out of 10) correct trials on two consecutive days.

###### Additional learning–massed and distributed sessions

After reaching criterion, the rats received 50 additional trials either massed within five consecutive days (10 trials per day for 5 days; Massed) or distributed across 10 weeks (weekly session of five trials; Distributed). In the Massed condition, the rats received surgery 5–10 days following the last training day (Recent) or 10–11 weeks following the last training day (Remote). Including these two criterion-to-surgery intervals aimed to dissociate the possibility of time-dependent consolidation processes (Squire et al., 2001). Rats in the Distributed condition received surgery 5–10 days after the last day of training. Thus, the criterion-to-surgery in this condition was identical to that of the Massed-Remote condition (10–11 weeks). The rats were assigned to either Sham or HPC surgery. Importantly, the rats were matched across groups according to S+ version (rubber duck and the red buoy), the number of days to reach criterion, and their average performance on the overtraining trials. Figure 1B illustrates the experimental design.

##### Surgery

The rats were anesthetized with isoflurane (Janssen, Toronto, ON, United States) in 0.8 L/min oxygen (Benson Medical Industries, Markham, ON, United States) at 14.7 PSIA at 21°C and given an analgesic (Metacam, 0.02 ml; 5 mg/mL, s.c.; Boehringer Ingelheim). The rats were then placed in a stereotaxic instrument (Kopf Instruments, Tujunga, CA, United States) and an incision was made along the midline of the scalp, exposing the skull. Eight holes were drilled in the skull above each hemisphere and the lesions were made by infusing *N*-methyl-D-aspartic acid (NMDA) (7.5 μg/μL in 0.9% saline, Sigma Chemical, St. Louis, MO, United States) into the HPC at 10 injection sites bilaterally. Injections were made using a 30-gauge injection needle attached to a 10 μl Hamilton syringe via polyethylene tubing (PE-50). NMDA was injected into the HPC at a rate of 0.4 μl/min for 45 s at each site with the exception of the last two ventral sites. For these sites the injection lasted 60 s. Following injection, the needle remained in place for an additional 2 min to facilitate the dispersion of NMDA into the HPC. Coordinates of the injection sites and the amount of NMDA injected at each site are summarized in Table 1. Following the NMDA injections, the incision was sutured and the rats were given a prophylaxis (diazepam 0.2–0.6 ml; 10 mg/ml, i.p.; Sabex, Boucherville, QC, Canada) to reduce seizure activity. Sham surgery was identical with the exception that no damage was done to the skull or brain of the rats. All the rats received an analgesic (Metacam, Oral Suspension 0.1 ml; 1.5 mg/mL, p.o.; Boehringer Ingelheim) daily for 7 days following surgery and were given 10–15 days of recovery before retention testing.

**TABLE 1:** Injection coordinates relative to bregma as well as volume of NMDA injected for complete NMDA lesions of the Hippocampus.

Anteroposterior (AP)	Mediolateral (ML)	Dorsoventral (DV)	Infusion Volume (μl)
−3.0	±1.0	−3.6	0.3
−3.0	±2.0	−3.6	0.3
−4.0	±2.0	−4.0	0.3
−4.0	±3.5	−4.0	0.3
−4.9	±3.0	−4.1	0.3
−4.9	±5.2	−5.0	0.3
−4.9	±5.2	−7.2	0.3
−5.7	±4.4	−4.4	0.3
−5.7	±5.1	−6.0	0.4
−5.7	±5.1	−7.3	0.4

##### Post-surgical retention and retraining

After recovering from surgery, the rats were tested for retention by receiving 10 discrimination trials that followed the same procedures as described for acquisition. The rats that performed below 80% accuracy during retention testing were given additional daily sessions of 10 trials until they reached 80% accuracy.

#### Histology

Following the completion of behavioral testing, the rats received an overdose of sodium pentobarbital (0.3 ml; 320 mg/ml, i.p. Schering Inc., Pointe-Claire, QC, Canada) and were perfused intracardially with 200 ml of phosphate buffered saline followed by 200 ml of 4% paraformaldehyde. The brains were extracted and stored in 4% paraformaldehyde for 24 h and then transferred to a 30% sucrose/0.1% sodium azide solution for a minimum of 24 h before being sectioned with a freezing microtome (American Optical Corporation, Buffalo, NY, United States) at a thickness of 40 μm. Every 12th section of the HPC was mounted on gelatin-coated glass slides and stained with cresyl violet. The stained sections were examined using a light microscope (Nikon Eclipse 80*i*; Nikon Instruments Inc., Melville, NY, United States) connected to a 1600 × 1200 megapixel digital camera (MicroFire; Optronics, Fremont, CA, United States) providing a live feed to a Dell Precision Computer. The extent of HPC damage was quantified using unbiased/assumption-free principles and the Cavalieri point-counting method (Mouton, 2002). Specifically, using the Stereologer 2000 program (Stereology Resource Center Inc., Chester, MD, United States), at a magnification of 2×, a systematic sampling grid with an area per point of 0.05 mm^2^ was randomly superimposed over each section. Points that contacted intact HPC cell fields (dentate gyrus and CA fields) were counted. The total number of points counted for each lesion brain was divided by the average number of points for five control rats (Mean = 635.3, SD = 55.49). This gave a proportion of remaining HPC tissue and the remainder of this was used as an estimate of HPC damage for each rat.

### Results

Four rats were excluded from the experiment before surgery due to failure to reach criterion on the visual discrimination. After 230 trials (23 training days) these rats were still performing at chance on the discrimination, which is 11 days beyond the average for the other rats. Thus, 34 rats (11 Massed-Recent, 11 Massed-Remote, and 12 Distributed) remained in the experiment.

#### Histology

**FIGURE 2:**
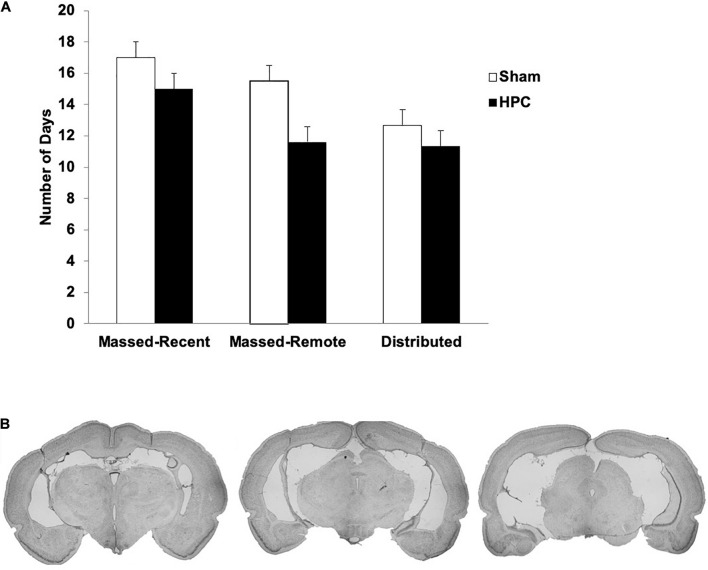
**(A)** Mean (+SEM) number of days to reach the learning criterion pre-surgically for Sham and HPC rats in the massed/recent, massed/remote, and distributed conditions. No statistical differences were found across groups, suggesting equivalent learning of the visual discrimination prior to the rats being given their overtraining trials. **(B)** Photomicrographs of coronal brain sections of a lesion rat that sustained 88.1% damage to the HPC. This lesion is representative of the average lesion size for rats across all experiments. Note that the lesions were made after criterion learning and the overtraining trials.

The average lesion size, as well as the smallest and largest lesion size, for each group is presented in Table 2 and photomicrographs of an average HPC lesion are found in Figure 2B. A one-way Analysis of Variance (ANOVA) found a statistically significant difference in the lesion sizes across groups, *F*(2, 13) = 12.14, *p* = 0.001. *Post hoc* tests revealed the lesions in the Massed-Remote group were significantly smaller than the lesions in the Massed-Recent or Distributed groups (*ps* < 0.05), whereas the lesions between the Massed-Recent and Distributed groups did not significantly differ in size.

**TABLE 2:** Descriptive Statistics of the HPC lesion size in each group of Experiment 1.

Group	HPC Damage (%)				
	*n*	Mean	Std. Dev.	Smallest	Largest
Recent	5	87.12	11.67	68.05	95.75
Remote	5	62.10	12.52	50.26	80.32
Distributed	6	88.96	4.01	83.00	94.81

Briefly, extensive neural death was found in all principle cell fields of the HPC (CA1–3, Dentate Gyrus; DG) of all lesion rats. In the Massed-Recent group, minimal sparing was found in the dorsal DG and CA3 of two rats and there was some sparing of the most posterior portion of the HPC in one rat. There was some damage extending into the posterior subiculum in three rats and the ventral subiculum in two rats. Additionally, one rat had minor unilateral damage to the entorhinal cortex. In the Massed-Remote group, very minimal sparing was found in the anterior portion of the HPC and considerably more sparing was found in the posterior portion. Specifically, the most posterior portion of the HPC was considerably spared in four rats. In the Distributed group, minimal sparing of the DG was observed bilaterally in three rats. There was some damage extending into the posterior subiculum bilaterally in two rats and unilaterally in one rat. Additionally, there was some minor damage to the entorhinal cortex unilaterally in one rat. In all groups, minor damage was sustained to the parietal cortex as a result of the cannulas being lowered and withdrawn during surgery.

#### Behavioral

##### Pre-operative training: reaching criterion

The average number of days to reach criterion pre-operatively across groups ranged between 11.33 and 15.5 days and is illustrated in Figure 2A. A 2 (surgery) × 3 (training condition) between-subjects ANOVA was performed to ensure that the groups were evenly matched on learning experience prior to surgery. No significant main effects for surgery group, *F*(1, 28) = 1.19, *p* = 0.29, or training condition, *F*(2, 28) = 1.12, *p* = 0.34, were found and there was no significant interaction between the two conditions, *F*(2, 28) = 0.12, *p* = 0.89. This suggests that, on average, the rats in all conditions were able to learn the visual discrimination within the same number of trials.

##### Post-operative testing: retention

**FIGURE 3:**
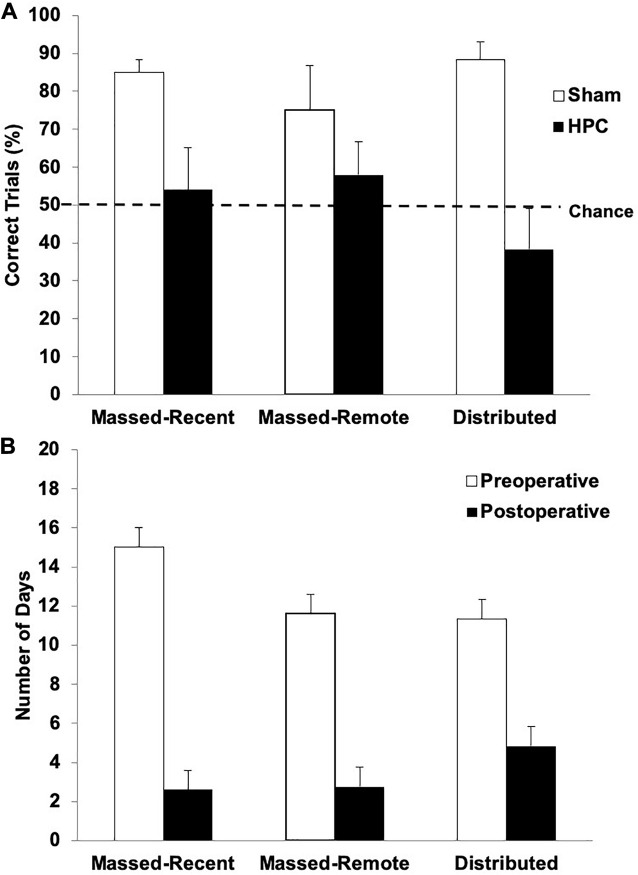
**(A)** Mean (+SEM) percent of correct trials during the post-surgical visual discrimination retention test for Sham and HPC rats in the massed/recent, massed/remote, and distributed conditions. Each Sham group showed a statistically significant bias (above 50%) in swimming to the S+ (*ps* < 0.05), suggesting that the control rats remembered the discrimination. In contrast, none of the HPC groups showed a preference for selecting the S+ (*ps* < 0.05) and showed fewer correct trials than the control rats (*p* < 0.05). Thus, the HPC rats, regardless of training condition, suffered from retrograde amnesia and the additional 50 training trials beyond criterion learning did not enable the memory to become more resistant to HPC damage. **(B)** Mean (+SEM) number of days for HPC rats in the massed/recent, massed/remote, and distributed conditions to relearn the visual discrimination compared to their pre-surgical performance (pre-surgical data also shown in Figure 2A). The lesion rats in all three conditions relearned the task in significantly fewer days than it took to learn the task initially (*p* < 0.05), suggesting that there was some memory trace remaining in non-HPC systems.

The percentage of correct trials across the retention session (10 trials) is illustrated in Figure 3A. One-tailed one-sample *t*-tests were conducted to determine whether the rats in each condition swam to the S+ on more trials than would be expected by chance (50%). The sham rats in all groups performed significantly above chance (Massed-Remote: *t*(5) = 2.13, *p* < 0.05; Massed-Recent: *t*(5) = 10.23, *p* < 0.05; Distributed: *t*(5) = 8.03, *p* < 0.05), suggesting they remembered the visual discrimination. The HPC rats in all three conditions, however, did not perform significantly above chance (Massed-Remote: *t*(4) = 0.93, *p* = 0.20; Massed-Recent: *t*(4) = 0.356, *p* = 0.37; Distributed: *t*(5) = −1.083, *p* = 0.16), suggesting that the rats with HPC damage did not remember the visual discrimination.

A 2 (surgery) × 3 (training condition) between-subjects ANOVA revealed a main effect of surgery, indicating that, overall, the rats with HPC damage performed significantly worse than control rats, *F*(1, 28) = 19.90, *p* < 0.05. Neither the main effect for training condition, *F*(2, 28) = 0.24, *p* = 0.79, or interaction between the surgery and training conditions, *F*(2, 28) = 1.74, *p* = 0.19, were statistically significant, indicating that the rats with HPC damage performed significantly worse than control rats, regardless of training condition.

##### Post-operative testing: reacquisition

Figure 3B illustrates the average number of days it took the HPC rats in each training condition to reach criterion both pre- and post-operatively. One rat was unable to relearn the visual discrimination post-operatively and was thus excluded from the reacquisition analysis. After 15 days of retraining (11 days beyond the average for the other rats), this rat was still performing no better than chance and was swimming to the right quadrant on every trial. With the exception of this rat, all the HPC rats were able to relearn the visual discrimination post-operatively. A mixed factorial ANOVA with between-subjects variable condition (Massed-Remote, Massed-Recent, and Distributed) and within-subjects variable time-of-learning (Pre-operative, Post-operative) was conducted on number of days to criterion in the HPC rats and revealed only a main effect of time-of-learning, *F*(1, 30) = 85.22, *p* = 0.001, indicating that it took HPC rats significantly fewer days to relearn the visual discrimination post-operatively than it took them to initially learn the discrimination. There was no main effect for condition *F*(2, 30) = 0.52, *p* = 0.6, and no interaction between condition and time-of-learning *F*(2, 30) = 1.73, *p* = 0.19, indicating that this pattern was true regardless of training condition.

### Discussion

The main objective of the current experiment was to assess whether additional learning beyond criterion and distributed over several weeks would make a visual discrimination memory more resistant to extensive HPC damage. During the retention test, the Sham rats showed a significant preference for the S+, suggesting they remembered the visual discrimination. In contrast, giving the rats 50 additional discrimination trials after reaching criterion, whether massed in 5 days or distributed over 10 weeks, did not facilitate the visual discrimination becoming independent of the HPC. In all instances, the HPC rats failed to show the same preference as the control rats, suggesting that HPC damage caused retrograde amnesia regardless of learning condition. These findings are consistent with two other studies demonstrating that the HPC is involved in long-term visual discrimination memory (Sutherland et al., 2001; Epp et al., 2008). Although there is evidence that overtraining can make a memory resistant to HPC damage in other tasks (Lehmann et al., 2009; Sutherland et al., 2010; Lehmann and McNamara, 2011), under the parameters of the present experiment, this did not extend to a visual discrimination memory. The second objective of the current experiment was to assess and control for the possibility of long-term memory systems consolidation, whereby the visual discrimination memory could have become independent of the HPC because of processes beyond the additional training. Despite extending the learning-to-lesion interval from 10 days (recent) to 10 weeks (remote), damaging the HPC had equal retrograde amnesic effects. These findings fail to support long-term systems consolidation views and this issue will be discussed in greater depth in the general discussion.

The HPC rats in the current experiment, despite suffering from retrograde amnesia, were able to quickly relearn the visual discrimination. This is consistent with evidence suggesting that HPC damage does not cause anterograde amnesia for this type of memory (Sutherland et al., 2001; Epp et al., 2008). Importantly, this also suggests that non-HPC systems can indeed support a visual discrimination memory. Despite continued distributed training beyond criterion, in the intact brain, the HPC seems to robustly overshadow and prevent the non-HPC systems from establishing a strong enough memory to independently support performance. It is possible that HPC overshadowing cannot be abated in this task, meaning that the non-HPC memory systems are unable to acquire a visual discrimination memory in the presence of the HPC. Alternatively, it is possible that the 50 trials beyond criterion in the current experiment were insufficient to strengthen the memory in the non-HPC memory systems and that even more trials may be required to mitigate the HPC overshadowing.

## Experiment 2

The evidence suggesting a lack of anterograde amnesia for visual discriminations comes from studies in which the rats had pre-operative experience in the task and initial learning occurred with the HPC intact (Driscoll et al., 2005; Epp et al., 2008). Thus, it is unknown whether rats with complete HPC damage are able to quickly learn a visual discrimination task if they have had no prior experience. This may provide insight into how many additional trials are needed to mitigate HPC overshadowing. Accordingly, in a second experiment we assessed the number of trials experimentally naïve HPC-damaged rats required to learn a visual discrimination. To address this question, experimentally naive rats received either complete damage to the HPC or sham surgery *before* being trained on a visual discrimination up to criterion.

### Methods

#### Subjects

Eleven male Long Evans rats served as subjects and were housed under the same conditions as described for Experiment 1.

#### Procedures

The rats received Sham or HPC surgery *before* the beginning of behavioral testing. The surgical procedures were identical to those described in Experiment 1 and the rats were given 10–15 days of recovery from surgery before the beginning of behavioral testing. The training in this experiment followed the *Criterion Training* procedures described in Experiment 1. The rats were euthanized the day after reaching criterion on the visual discrimination (80% accuracy on two consecutive days).

### Results

#### Histology

The NMDA injections resulted in an average of 86.3% damage to the HPC (SD = 8.6%; smallest lesion = 75.44%, largest = 94.96%). There was substantial damage to the HPC in all cell fields (CA1–3, DG), however some minor sparing was found in the dorsal HPC of five rats bilaterally and in the ventral HPC of four rats bilaterally and two rats unilaterally. In addition, some damage was sustained to the posterior subiculum in three rats bilaterally and one rat unilaterally. All rats also sustained minor damage to the parietal cortex as a result of the injection cannulae.

#### Behavioral

##### Post-operative acquisition

**FIGURE 4:**
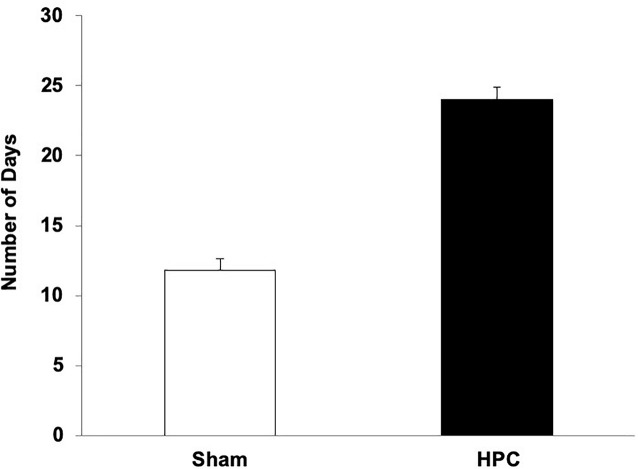
Mean (+SEM) number of days for naïve Sham and HPC rats to learn the visual discrimination after their surgery. The rats with HPC lesions took twice as many days to learn the visual discrimination as control rats (*p* < 0.05), suggesting anterograde amnesia and that the non-HPC systems need many trials to acquire a visual discrimination.

The average number of days to reach criterion for the HPC and Sham rats is illustrated in Figure 4. An independent *t*-test revealed that the HPC rats took significantly longer to learn the visual discrimination than the Sham rats, *t*(9) = −4.42, *p* < 0.05.

### Discussion

Rats with HPC damage, naïve to the visual discrimination task demands, took twice as much training as control rats to learn the discrimination, suggesting that the lesions caused anterograde amnesia. Although previous studies report intact learning following HPC damage in this task (Driscoll et al., 2005; Epp et al., 2008), it appears that this is only the case when the HPC rats have had prior visual discrimination experience. In this task, procedural aspects must be acquired (e.g., search for a platform) as well as properties of the stimuli, and the association of the S+ with the escape. It is unclear which learning and memory aspects were affected by the lesions. The current findings, however, still confirm that the non-HPC systems are capable of acquiring and retaining a visual discrimination memory, but that these systems, in the naïve rat, needs substantially more training than described previously.

## Experiment 3

In Experiment 1, giving the rats 50 additional discrimination trials, after reaching criterion, did not make a visual discrimination memory resistant to post-training HPC damage. One possibility accounting for this outcome is that the non-HPC memory systems require many more discrimination trials to acquire an independent visual discrimination memory. This possibility is corroborated by the findings of Experiment 2, suggesting that more than 200 trials are needed to train the experimentally naïve non-HPC systems. Therefore, in this experiment we increased the overtraining following criterion learning from 50 to 230 distributed trials (10 trials in one session every 3 days for 10 weeks) in order to assess whether this change would make the visual discrimination resistant to HPC damage.

### Methods

#### Subjects

Nineteen male Long Evans rats served as subjects in this experiment and they were housed under the same conditions as previously described.

#### Procedures

All behavioral and surgical procedures were identical to those described in Experiment 1 with the exception that after reaching criterion, the rats received an additional 230 discrimination trials (10 trials every 3 days for 10 weeks). The rats received HPC lesions or sham surgery 5–10 days following the last day of training.

### Results

#### Histology

The NMDA injections resulted in an average of 85.3% damage to the HPC (SD = 7.6%; smallest = 67.6%, largest = 94.3%). In all rats, there was substantial damage to all HPC cell fields (CA1–3, DG). There was very little, if any, sparing in the anterior portion of the HPC and considerably more sparing in the posterior portion for all rats. In seven rats, there was additional damage extending into the posterior subiculum, bilaterally for five rats and unilaterally for two rats. All rats sustained minor damage to the parietal cortex as a result of the injection cannulas being lowered and withdrawn during surgery.

#### Behavioral

##### Pre-operative training: criterion training

An independent *t*-test comparing Sham and HPC lesion rats revealed no significant difference between the surgery groups on the number of days required to reach the learning criterion [Sham (*M* = 13.5, SEM = 1.79) and HPC (*M* = 13.29, SEM = 3.16); *t*(17) = −0.064, *p* = 0.95]. This suggests that that the pre-operative discrimination experience was comparable between both groups.

##### Post-operative testing: retention

**FIGURE 5:**
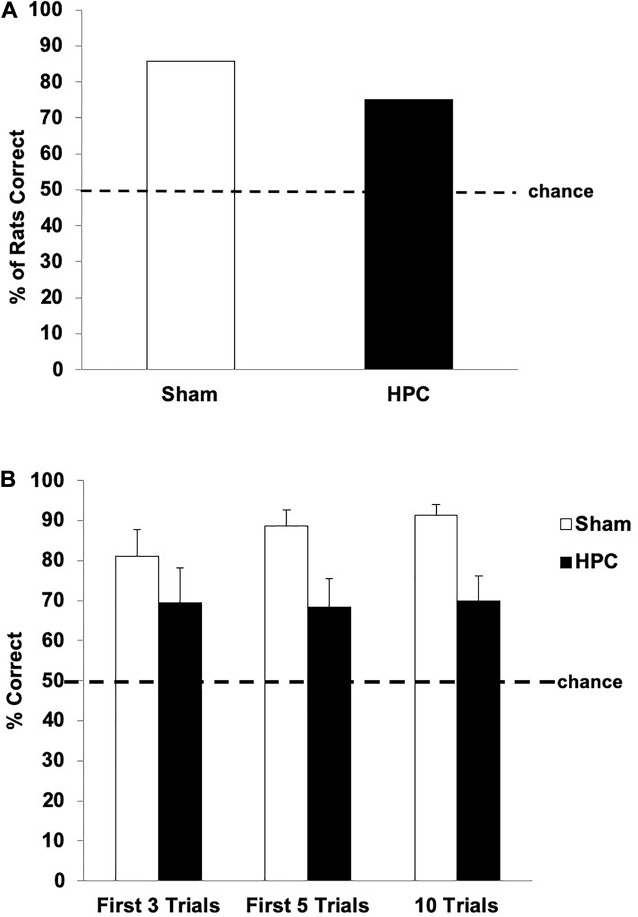
Post-surgical retention performance of the Sham and HPC rats that received 230 overtraining trials following the visual discrimination criterion learning. **(A)** Percent of rats in each group choosing S+ on the first retention trial. Although both groups tended to show a preference in swimming to the S+, the bias was not statistically significant for either group (*p* > 0.05). **(B)** Mean (±SEM) percent of correct trials on the first three trials, first five trials, and all 10 retention trials. Both Sham and HPC rats, regardless of the number of trials considered, showed a an S+ selection statically above chance (50%, *ps* < 0.05), suggesting that both groups remembered the discrimination. The HPC rats did selected the S+ fewer times than the Sham group across all 10 trials, but not earlier, suggesting that the control rats reacquired the discrimination and that the HPC did not show this relearning over the test. Overall, the 230 overlearning trials mitigated the amnesic effects of the HPC damage, but impaired fast reacquisition of the visual discrimination.

Figure 5 shows the retention data for both groups. One-sample *t*-tests indicated that both the Sham and HPC groups showed a significant preference for the S+ over the 10 retention trials (above 50%; Sham: *t*(6) = 15.88, *p* < 0.05; HPC: *t*(11) = 3.25, *p* < 0.05; Figure 5B), suggesting that both groups remembered the visual discrimination. A between group *t*-test, however, indicated that the HPC group selected the S+ significantly less than the Sham group over the 10-trial test, *t*(17) = 2.56, *p* < 0.05, suggesting that the memory was weaker and/or the HPC rats potentially quickly reacquired the discrimination. To assess this possibility, we examined the performance of both groups on the first retention trial, the first three, and the first five independently. On the first retention trial 85.71% of Sham and 75% of HPC rats correctly swam to the S+; though a chi-square test failed to detect a significant preference for the S+ for either group (50%; *ps* > 0.05; see Figure 5A). In contrast, the statistical analysis of performance across the first three and first five retention trials revealed that both groups remembered the discrimination, which is illustrated in Figure 5B. Specifically, one-sample *t*-tests indicated that both the Sham and HPC groups, in these two blocks of cumulative retention trials, showed a significant preference for the S+ (above 50%; *ps* < 0.05), suggesting the both groups remembered the visual discrimination. A between group *t*-test also indicated that the HPC group showed a S+ preference as strong as the Sham group over the first three trials, *t*(17) = 0.93, *p* = 0.36. Like for the total 10 trials findings, however, the HPC rats did not show as strong of a preference for the S+ when considering the first five trials, *t*(17) = 2.03, *p* < 0.05. Thus, the Sham group, but not the HPC group, seemed to benefit from some discrimination relearning during the test.

## General Discussion

Compelling evidence suggests that the HPC can interfere, in some way, with non-HPC systems, ultimately reducing their ability to acquire and support an independent memory–an interference process termed overshadowing (Packard et al., 1989; Sutherland et al., 2010; Lee et al., 2016). The present study sought to examine whether distributed learning would mitigate this overshadowing and enable a visual discrimination memory trace to become sufficiently established in non-HPC systems and to be resistant to HPC damage. In Experiment 1, 50 overtraining trials, after criterion learning, did not lead to a memory that was resistant to HPC damage. Whether the additional 50 trials were massed within a few days or distributed across 10 weeks, the lesions caused retrograde amnesia. The selection of 50 trials following criterion learning was based on prior studies demonstrating that rats with HPC damage can quickly reacquire a visual discrimination (Driscoll et al., 2005; Epp et al., 2008). In Experiment 2, however, we determined that experimentally naive rats with pre-training HPC lesions need more than 200 trials beyond those of the control group to learn a discrimination, meaning that visual discrimination learning in non-HPC systems, even after the removal of the interference, is substantially slower than we originally hypothesized. Therefore, in Experiment 3, we re-examined the possible mitigating effects of distributed overtraining on the overshadowing process by increasing the number post-criterion trials to 230. In this instance, lesions of the HPC failed to cause retrograde amnesia, demonstrating that a visual discrimination memory can indeed change from HPC-dependent to -independent, but only following numerous overtraining trials. Importantly, this also demonstrates that the overshadowing process or HPC-interference is not absolute and that with continued learning a representation can be strengthened in non-HPC systems to the extent that it no longer critically requires support from the HPC.

The inference that the HPC lesions did not cause retrograde amnesia following 230 overtraining trials in Experiment 3 comes from the early part of the 10-trial retention test. Whether the first retention trial, the first three, or the first five are considered, the selection bias of the HPC rats clearly suggests that they remembered the discrimination and did so as well as the control group. This is of importance because the early trials of the retention test are the least likely to be influenced by discrimination relearning and can be considered a “purer” index of memory. When analyzing performance on the entire 10 trial block, however, the selection bias suggests that the HPC rats remembered the discrimination but not as strongly as the control rats. This difference seems to be due to a ubiquitous performance of the control group over the test, with a weaker bias for the S+ at the beginning of the test in comparison the entire test. Thus, the control group seems to have benefited slightly from relearning over the 10 trials, whereas the HPC group’s performance remained stable. Nevertheless, the overtraining trials in Experiment 3 resulted in a memory trace in non-HPC systems that could be expressed without the HPC. It is also important to note that it is improbable that the spared memory in Experiment 3 is a result of insufficient damage to the HPC. In all three experiments the neurotoxic lesions produced substantial HPC damage (over 80% in each experiment) and, in Experiment 1, lesions comparable and even smaller lesions than those in Experiment 3 were sufficient to cause retrograde amnesia. Therefore, we conclude that the successful visual discrimination performance of the HPC rats in Experiment 3 was supported by a trace in non-HPC systems resulting form the overtraining.

The development of an independent visual discrimination memory in non-HPC systems with additional learning has significant theoretical implications and supports the DRT view. Overtraining trials resulting in a memory that typically requires the HPC to become more strongly established in other networks is consistent with the DRT. Critically, this non-HPC trace increase also made the memory resistant to HPC damage. These imply that there are at least two systems that can support a visual discrimination memory. Evidence of the involvement of the HPC system in visual discrimination memory also comes from studies that found that post-training damage to this system causes retrograde amnesia (Sutherland et al., 2001; Driscoll et al., 2005; Epp et al., 2008), a finding that we replicated in Experiment 1. The evidence of the involvement of other systems comes from Experiments 1 and 2 as well as other studies (Driscoll et al., 2005; Epp et al., 2008) showing that in absence of the HPC rats are able to learn or reacquire a visual discrimination, which is only feasible if supported by non-HPC systems. Importantly, this study is the first to demonstrate that the non-HPC systems can acquire a visual discrimination memory with the HPC intact (Experiment 3). Combined our findings are consistent with the three major tenets of the DRT: multiple memory systems, HPC overshadowing, and mitigation of the overshadowing with additional learning (Sutherland et al., 2010).

In the current study, removing the HPC overshadowing, or interference, did not promote fast independent acquisition of the visual discrimination in the other systems. This contrasts with the typical HPC overshadowing observations in other tasks, such as contextual fear conditioning (Wiltgen et al., 2006; Lehmann et al., 2009), novel object preference (Gaskin et al., 2003), shock-probe conditioning (Lehmann et al., 2005, 2006), home base memory (Travis et al., 2010), cued place memory (Ramos, 2013), and fear-potentiated startle (Lehmann et al., 2010), in which HPC-damaged rats learn as quickly as control rats. It is unclear why non-HPC systems are slow in independently acquiring and retaining visual discrimination information versus other memories that usually require the HPC. Perhaps, in naïve rats, the early phase of visual discrimination learning in the pool involves more procedural learning (e.g., swimming to a platform, approaching a floating object leads to an escape possibility) than in the other tasks. Only once the procedural components are mastered could learning the S+ and S− associations more rapidly proceed. This possibility would be consistent with Epp et al.’s (2008) findings that experienced HPC-damaged rats, those that have mastered the task demands, can rapidly learn a new visual discrimination. Despite the overshadowing distinction between the present study and that of others, the visual discrimination overtraining still engaged and strengthened the memory trace beyond the HPC.

There are several other demonstrations that additional learning can reduce the dependency of a memory on the HPC in rats and they suggest that the phenomenon is not only specific to visual discriminations (Winocur et al., 2005; Lehmann et al., 2009; Sutherland et al., 2010; Lehmann and McNamara, 2011; Shepherd et al., 2021). The current findings add to those reported in contextual fear conditioning studies, in which distributing the conditioning over 10–11 sessions rather than one massed session prevents the retrograde amnesic effect of HPC damage (Lehmann et al., 2009; Shepherd et al., 2021). Additionally, object recognition overtraining strengthens the memory trace beyond the HPC in the novel object preference task (Sutherland et al., 2010) and rearing rats in a complex environment promotes the formation of a HPC-independent spatial memory trace (Winocur et al., 2005). Therefore, overtraining and/or distributed learning parameters lead to non-HPC memory network modifications to the extent that the HPC is no longer required for several types of mnemonic information. The network supporting the memory that is HPC-independent after additional learning likely differs, at least in part, across each type of memory. Also, the memory trace leading to successful recall and expression from the non-HPC system is unlikely a duplicate of the trace in the HPC (Lee et al., 2016). Rather we argue that interactive HPC and non-HPC systems, as evidenced by the overshadowing, are involved from the start and that each structure or node within the systems have idiosyncratic contributions to an overall trace. Overtraining or re-experiencing the learning event increases the strength of the trace in and across its various nodes and systems. The strengthening would involve plastic changes supported by cellular consolidation processes brought about with each new learning episode (Lee, 2008; Dudai, 2012). After sufficient strengthening of the trace, recall could be achieved with a subset of the nodes and systems. Hence, after HPC damage, some mnemonic information specific to this structure, such as configural associations (Rudy and Sutherland, 1995), would be lost, but other mnemonic information from the other nodes would now suffice for successful behavioral expression.

Current long-term systems consolidation theories cannot fully account for the retrograde amnesia findings we observed in Experiment 1 or the spared memory in Experiment 3. There are two main long-term memory system consolidation views: (1) the Standard Model of Consolidation and (2) the Multiple Trace Theory also now termed the Memory Transformation Theory. Both theories suggest that memory undergoes strengthening in neocortical structures resulting in a memory that no longer requires the HPC (Squire, 1992; Alvarez and Squire, 1994; Squire and Alvarez, 1995; Nadel and Moscovitch, 1997; Frankland and Bontempi, 2005; Winocur et al., 2010; Sekeres et al., 2018). In the Standard Model, the neocortical memory incorporates all qualitative aspects of the one that originally required the HPC (Squire, 1992; Alvarez and Squire, 1994; Squire and Alvarez, 1995; Frankland and Bontempi, 2005). In contrast, the Multiple Trace/Transformation Theory suggests that the memory that becomes independent of the HPC is qualitatively different (Nadel and Moscovitch, 1997; Winocur et al., 2010; Sekeres et al., 2018). The memory in the neocortical network would be semantic/gist like, whereas any episodic information from the original event would always remain dependent on the HPC. Both theories suggest that the development of the HPC-independent memory occurs because of HPC-neocortical interactions that would likely occur during online (e.g., recall) and offline (e.g., sleep) processes. With a period providing sufficient interactions between memory acquisition and the onset of HPC damage, the memory should have consolidated in the neocortex and have become HPC independent. In Experiment 1 of the current study, providing a 10-week interval between the criterion learning and the HPC lesions did not result in any evidence of a spared visual discrimination memory. Indeed, the performance of the HPC-Massed-Remote group was at chance, no better than the HPC-Massed-Recent group, and significantly worse than its respective control group. Moreover, adding 50 trials beyond criterion, five a week for the 10-week period, still did not prevent the retrograde amnesic effects of the lesions (HPC-Distributed). Thus, the learning-to-lesion period, which was more than double the period manipulated in many studies that have reported temporally graded amnesia and evidence of systems consolidation in rats (Kim and Fanselow, 1992; Winocur et al., 2001, 2013; Wang et al., 2009; Wiltgen et al., 2010), was clearly insufficient to make a visual discrimination memory trace HPC independent. A visual discrimination memory resistant to HPC damage was only achieved when the rats received 10 trials every 3 days over the 10 weeks that followed criterion learning. This overtraining may have promoted more interaction bouts between the HPC and non-HPC systems to promote systems consolidation. More parsimoniously, however, the overtraining sessions most likely strengthened the trace in its various nodes and systems as we suggested earlier. For example, the overtraining may have additively strengthened a striatal stimulus response association (Packard et al., 1989; McDonald and White, 1993) to a point that this type of memory supported successful behavioral performance of the HPC rats during the retention test in Experiment 3. This would be consistent with the evidence suggesting that the HPC system can dominate over other memory systems, including the striatal one (Packard et al., 1989). It is also consistent with the finding from Packard and McGaugh (1996) that continued navigation training in a cross-maze task can lead to a shift from a HPC-dependent to a striatal-dependent performance strategy. Although we propose here that a different form of memory is supporting the HPC rats’ retention performance, at no point do we suggest a transformation. Rather, each system was strengthened with each new overtraining session and not because the HPC interacted with the striatum more during the learning-to-lesion interval, a position consistent with the DRT (Sutherland et al., 2010).

In conclusion, overtraining alters the trace that supports a memory. Ebbinghaus was amongst the first, if not the first, to describe this phenomenon, whether by mitigating forgetting or extinction (Ebbinghaus, 1964). Yet, the trace changes at the neural systems level that accompany the memory strengthening with overtraining has gained poor consideration. Here we show that a memory that typically requires the HPC can change to no longer require the HPC with additional training, meaning that overtraining made a memory become HPC independent. This change in contribution of the HPC has important implication for theories of long-term memory organization with increased support for the DRT.

## Data Availability Statement

The raw data supporting the conclusions of this article will be made available by the authors, without undue reservation.

## Ethics Statement

The animal study was reviewed and approved by Trent University Animal Care Committee.

## Author Contributions

HL and MG conceived the idea. HL designed the experiment. MS collected the data. HL and MS analyzed the data. All authors contributed to the writing of the manuscript.

## Conflict of Interest

The authors declare that the research was conducted in the absence of any commercial or financial relationships that could be construed as a potential conflict of interest.

## Publisher’s Note

All claims expressed in this article are solely those of the authors and do not necessarily represent those of their affiliated organizations, or those of the publisher, the editors and the reviewers. Any product that may be evaluated in this article, or claim that may be made by its manufacturer, is not guaranteed or endorsed by the publisher.

## References

[B1] Alvarez P. Squire L. R. (1994). Memory consolidation and the medial temporal lobe: a simple network model. *Proc. Natl. Acad. Sci. U.S.A.* 91 7041–7045. 10.1073/pnas.91.15.7041 8041742 PMC44334

[B2] Bahrick H. P. Phelphs E. (1987). Retention of Spanish vocabulary over 8 years. *J. Exp. Psychol. Learn. Mem. Cogn.* 13 344–349. 10.1037/0278-7393.13.2.344

[B3] Broadbent N. J. Clark R. E. (2013). Remote context fear conditioning remains hippocampus-dependent irrespective of training protocol, training-surgery interval, lesion size, and lesion method. *Neurobiol. Learn. Mem.* 106 300–308. 10.1016/j.nlm.2013.08.008 23994542

[B4] Driscoll I. Howard S. R. Prusky G. T. Rudy J. W. Sutherland R. J. (2005). Seahorse wins all races: hippocampus participates in both linear and non-linear visual discrimination learning. *Behav. Brain Res.* 164 29–35. 10.1016/j.bbr.2005.05.006 16024101

[B5] Dudai Y. (2012). The restless engram: consolidations never end. *Annu. Rev. Neurosci.* 35 227–247. 10.1146/annurev-neuro-062111-150500 22443508

[B6] Ebbinghaus H. (1964). *Memory: A Contribution To Experimental Psychology.* New York, NY: Dover Publications. 10.5214/ans.0972.7531.200408 PMC4117135 25206041

[B7] Epp J. Keith J. R. Spanswick S. C. Stone J. C. Prusky G. T. Sutherland R. J. (2008). Retrograde amnesia for visual memories after hippocampal damage in rats. *Learn. Mem.* 15 214–221. 10.1101/lm.788008 18385476 PMC2327263

[B8] Fanselow M. S. Poulos A. M. (2004). The Neuroscience of mammalian associative learning. *Annu. Rev. Psychol.* 56 207–234. 10.1146/annurev.psych.56.091103.070213 15709934

[B9] Frankland P. W. Bontempi B. (2005). The organization of recent and remote memories. *Nat. Rev. Neurosci.* 6 119–130. 10.1038/nrn1607 15685217

[B10] Gaskin S. Tremblay A. Mumby D. G. (2003). Retrograde and anterograde object recognition in rats with hippocampal lesions. *Hippocampus* 13 962–969. 10.1002/hipo.10154 14750658

[B11] Kim J. J. Fanselow M. S. (1992). Modality-specific retrograde amnesia of fear. *Science* 256 675–677. 10.1126/science.1585183 1585183

[B12] Lee J. L. (2008). Memory reconsolidation mediates the strengthening of memories by additional learning. *Nat. Neurosci.* 11 1264–1266. 10.1038/nn.2205 18849987

[B13] Lee J. Q. Zelinski E. L. McDonald R. J. Sutherland R. J. (2016). Heterarchic reinstatement of long-term memory: a concept on hippocampal amnesia in rodent memory research. *Neurosci. Biobehav. Rev.* 71 154–166. 10.1016/j.neubiorev.2016.08.034 27592152

[B14] Lehmann H. McNamara K. C. (2011). Repeatedly reactivated memories become more resistant to hippocampal damage. *Learn. Mem.* 18 132–135. 10.1101/lm.2000811 21325434

[B15] Lehmann H. Carfagnini A. Yamin S. Mumby D. G. (2005). Context-dependent effects of hippocampal damage on memory in the shock-probe test. *Hippocampus* 15 18–25. 10.1002/hipo.20024 15390168

[B16] Lehmann H. Lacanilao S. Sutherland R. J. (2007). Complete or partial hippocampal damage produces equivalent retrograde amnesia for remote contextual fear memories. *Eur. J. Neurosci.* 25 1278–1286. 17355254 10.1111/j.1460-9568.2007.05374.x

[B17] Lehmann H. Lecluse V. Houle A. Mumby D. (2006). Retrograde amnesia following hippocampal lesions in the shock-probe conditioning test. *Hippocampus* 16 379–387. 10.1002/hipo.20159 16411184

[B18] Lehmann H. Rourke B. K. Booker A. Glenn M. J. (2013). Single session contextual fear conditioning remains dependent on the hippocampus despite an increase in the number of context-shock pairings during learning. *Neurobiol. Learn. Mem.* 106 294–299. 10.1016/j.nlm.2012.10.011 23142771

[B19] Lehmann H. Sparks F. T. O’Brien J. McDonald R. J. Sutherland R. J. (2010). Retrograde amnesia for fear-potentiated startle in rats after complete, but not partial, hippocampal damage. *Neuroscience* 167 974–984. 10.1016/j.neuroscience.2010.03.005 20226233

[B20] Lehmann H. Sparks F. T. Spanswick S. C. Hadikin C. McDonald R. J. Sutherland R. J. (2009). Making context memories independent of the hippocampus. *Learn. Mem.* 16 417–420. 10.1101/lm.1385409 19553378 PMC2704104

[B21] Maren S. Aharonov G. Fanselow M. S. (1997). Neurotoxic lesions of the dorsal hippocampus and pavlovian fear conditioning in rats. *Behav. Brain Res.* 88 261–274. 10.1016/S0166-4328(97)00088-0 9404635

[B22] McDonald R. J. White N. M. (1993). A triple dissociation of memory systems: hippocampus, amygdala, and dorsal striatum. *Behav. Neurosci.* 107 3–22. 10.1037/0735-7044.107.1.3 8447956

[B23] Milner B. (1959). The memory defect in bilateral hippocampal lesions. *Psychiatr. Res. Rep. Am. Psychiatr. Assoc.* 11 43–58. 14422670

[B24] Mouton P. R. (2002). *Principles and Practices of Unbiased Stereology: An Introduction for Bioscientists.* Baltimore, MD: The Johns Hopkins University Press.

[B25] Nadel L. Moscovitch M. (1997). Memory consolidation, retrograde amnesia and the hippocampal complex. *Curr. Opin. Neurobiol.* 7 217–227. 10.1016/S0959-4388(97)80010-4 9142752

[B26] Packard M. G. McGaugh J. L. (1996). Inactivation of hippocampus or caudate nucleus with lidocaine differentially affects expression of place and response learning. *Neurobiol. Learn. Mem.* 65 65–72. 10.1006/nlme.1996.0007 8673408

[B27] Packard M. G. Hirsh R. White N. M. (1989). Differential effects of fornix and caudate nucleus lesions on two radial maze tasks: evidence for multiple memory systems. *J. Neurosci.* 9 1465–1472. 10.1523/JNEUROSCI.09-05-01465.1989 2723738 PMC6569845

[B28] Ramos J. M. J. (2013). Profound retrograde but absence of anterograde amnesia for cued place learning in rats with hippocampal lesions. *Behav. Brain Res.* 236 102–109. 10.1016/j.bbr.2012.08.036 22944137

[B29] Rempel-Clower N. L. Zola S. M. Squire L. R. Amaral D. G. (1996). Three cases of enduring memory impairment after bilateral damage limited to the hippocampal formation. *J. Neurosci.* 16 5233–5255. 10.1523/JNEUROSCI.16-16-05233.1996 8756452 PMC6579309

[B30] Rudy J. W. Sutherland R. J. (1995). Configural association theory and the hippocampal formation: an appraisal and reconfiguration. *Hippocampus* 5 375–389. 10.1002/hipo.450050502 8773252

[B31] Scoville W. B. Milner B. (1957). Loss of recent memory after bilateral hippocampal lesions. *J. Neurochem.* 20 11–21. 10.1136/jnnp.20.1.11 13406589 PMC497229

[B32] Sekeres M. J. Winocur G. Moscovitch M. Anderson J. A. E. Pishdadian S. Martin Wojtowicz J. (2018). Changes in patterns of neural activity underlie a time-dependent transformation of memory in rats and humans. *Hippocampus* 28 745–764. 10.1002/hipo.23009 29989271

[B33] Shepherd E. H. Fournier N. M. Sutherland R. J. Lehmann H. (2021). Distributed learning episodes create a context fear memory outside the hippocampus that depends on perirhinal and anterior cingulate cortices. *Learn. Mem.* 28, 405–413. 10.1101/lm.053396.121 34663693 PMC8525424

[B34] Sherry D. F. Schacter D. L. (1987). The evolution of multiple memory systems. *Psychol. Rev.* 94 439–454. 10.1037/0033-295X.94.4.439

[B35] Sparks F. T. Spanswick S. C. Lehmann H. Sutherland R. J. (2013). Neither time nor number of context-shock pairings affect long-term dependence of memory on hippocampus. *Neurobiol. Learn. Mem.* 106 309–315. 10.1016/j.nlm.2013.05.008 23747567

[B36] Squire L. R. (1992). Memory and the hippocampus: a synthesis from findings with rats, monkeys, and humans. *Psychol. Rev.* 99 195–231. 10.1037/0033-295x.99.2.195 1594723

[B37] Squire L. R. Alvarez P. (1995). Retrograde amnesia and memory consolidation: a neurobiological perspective. *Curr. Opin. Neurobiol.* 5 169–177. 10.1016/0959-4388(95)80023-9 7620304

[B38] Squire L. R. Zola S. M. (1996). Memory, memory impairment, and the medial temporal lobe. *Cold Spring Harb. Symp. Quant. Biol.* 61 185–195. 10.1101/SQB.1996.061.01.021 9246447

[B39] Squire L. R. Clark R. E. Knowlton B. J. (2001). Retrograde amnesia. *Hippocampus* 11 50–55. 10.1002/1098-1063(2001)11:1<50::AID-HIPO1019>3.0.CO;2-G 11261772

[B40] Sutherland R. J. Lehmann H. (2011). Alternative conceptions of memory consolidation and the role of the hippocampus at the systems level in rodents. *Curr. Opin. Neurobiol.* 21 446–451. 10.1016/j.conb.2011.04.007 21592780

[B41] Sutherland R. J. Lehmann H. Spanswick S. C. Sparks F. T. Melvin N. R. (2006). Growth points in research on memory and hippocampus. *Can. J. Exp. Psychol.* 60 166–174. 10.1037/cjep20060016 17133891

[B42] Sutherland R. J. O’Brien J. Lehmann H. (2008). Absence of systems consolidation of fear memories after dorsal, ventral, or complete hippocampal damage. *Hippocampus* 18 710–718. 10.1002/hipo.20431 18446823

[B43] Sutherland R. J. Sparks F. T. Lehmann H. (2010). Hippocampus and retrograde amnesia in the rat model: a modest proposal for the situation of systems consolidation. *Neuropsychologia* 48 2357–2369. 10.1016/j.neuropsychologia.2010.04.015 20430043 PMC2900526

[B44] Sutherland R. J. Weisend M. P. Mumby D. Astur R. S. Hanlon F. M. Koerner A. (2001). Retrograde amnesia after hippocampal damage: recent vs. remote memories in two tasks. *Hippocampus* 11 27–42. 10.1002/1098-1063(2001)11:1<27::AID-HIPO1017>3.0.CO;2-4 11261770

[B45] Travis S. G. Sparks F. T. Arnold T. Lehmann H. Sutherland R. J. Whishaw I. Q. (2010). Hippocampal damage produces retrograde but not anterograde amnesia for a cued location in a spontaneous exploratory task in rats. *Hippocampus* 20 1095–1104. 10.1002/hipo.20710 19957337

[B46] Wagner A. R. Rudy J. W. Whitlow J. W. (1973). Rehearsal in animal conditioning. *J. Exp. Psychol.* 97 407–426. 10.1037/h0034136 4705247

[B47] Wang S. H. Teixeira C. M. Wheeler A. L. Frankland P. W. (2009). The precision of remote context memories does not require the hippocampus. *Nat. Neurosci.* 12 253–255. 10.1038/nn.2263 19182794

[B48] White N. M. McDonald R. J. (2002). Multiple parallel memory systems in the brain of the rat. *Neurobiol. Learn. Mem.* 77 125–184. 10.1006/nlme.2001.4008 11848717

[B49] Wiltgen B. J. Sanders M. J. Anagnostaras S. G. Sage J. R. Fanselow M. S. (2006). Context fear learning in the absence of the hippocampus. *J. Neurosci.* 26 5484–5491. 10.1523/JNEUROSCI.2685-05.2006 16707800 PMC6675287

[B50] Wiltgen B. J. Zhou M. Cai Y. Balaji J. Karlsson M. G. Parivash S. N. (2010). The Hippocampus plays a selective role in the retrieval of detailed contextual memories. *Curr. Biol.* 20 1336–1344. 20637623 10.1016/j.cub.2010.06.068 PMC2928141

[B51] Winocur G. McDonald R. M. Moscovitch M. (2001). Anterograde and retrograde amnesia in rats with large hippocampal lesions. *Hippocampus* 11 18–26. 10.1002/1098-1063(2001)11:1<18::AID-HIPO1016>3.0.CO;2-5 11261769

[B52] Winocur G. Moscovitch M. Bontempi B. (2010). Memory formation and long-term retention in humans and animals: convergence towards a transformation account of hippocampal-neocortical interactions. *Neuropsychologia* 48 2339–2356. 10.1016/j.neuropsychologia.2010.04.016 20430044

[B53] Winocur G. Moscovitch M. Fogel S. Rosenbaum R. S. Sekeres M. (2005). Preserved spatial memory after hippocampal lesions: effects of extensive experience in a complex environment. *Nat. Neurosci.* 8 273–275. 10.1038/nn1401 15723062

[B54] Winocur G. Sekeres M. J. Binns M. A. Moscovitch M. (2013). Hippocampal lesions produce both nongraded and temporally graded retrograde amnesia in the same rat. *Hippocampus* 23 330–341. 10.1002/hipo.22093 23401223

